# The complex conundrum of Merkel cell carcinoma cellular ancestry

**DOI:** 10.1038/s41419-025-07892-7

**Published:** 2025-07-29

**Authors:** Chiara Mazziotta, Fernanda Martini, John Charles Rotondo

**Affiliations:** 1 Department of Medical Sciences, University of Ferrara, 44121 Ferrara, Italy; 2 Center for Studies on Gender Medicine - Department of Medical Sciences, University of Ferrara, 64/b, Fossato di Mortara Street, Ferrara, 44121 Italy; 3 Department of Medical Oncology, Dana-Farber Cancer Institute, Harvard Medical School, Boston, Massachusetts USA; 4 Laboratory for Technologies of Advanced Therapies (LTTA), University of Ferrara, 64/b, Fossato di Mortara Street, 44121 Ferrara, Italy; 5 IRCCS Ospedale Policlinico San Martino, Largo Rosanna Benzi, 10, 16132 Genova, Italy

**Keywords:** Oncogenesis, Skin cancer

## Abstract

Merkel cell carcinoma (MCC) is a rare but lethal skin neoplasm, caused, in approximately 80% of cases, by the genomic integration of Merkel cell polyomavirus (MCPyV) and the expression of viral oncoproteins small T (sT) and large T (LT) antigens. Virus-negative MCCs exhibit extensive UV-induced mutations. Although there is a growing understanding of MCC pathogenesis, the cellular origin of MCC remains a topic of intense investigation and debate. In this perspective, we will provide a description and discussion of the current theories regarding the cellular ancestry of MCC. The most recent findings point in favor of a potential epithelial origin of MCC. MCPyV integration likely occurs in an epithelial precursor cell prior to MCPyV-driven clonal expansion, while the same originating cell type may undergo a specific molecular switch that drives neuroendocrine differentiation, leading to UV-mutated, virus-negative MCCs. Identifying the cellular origin of MCC is crucial for developing accurate pre-clinical models and advancing clinical applications.

## Merkel Cell Carcinoma

Merkel cell carcinoma (MCC) is a highly malignant tumor with the highest mortality rate among cutaneous tumors, estimated at ~35% within 5 years. Approximately 12% of MCC patients present metastatic/distant disease at diagnosis, which is associated with a survival rate of ~14%. In ~80% of MCC cases, oncogenesis involves the integration of Merkel cell polyomavirus (MCPyV) DNA into the host genome, the expression of viral oncoproteins small T and large T antigens (sT and LT), and LT truncation (tLT). In particular, tLT binds to and inhibits the protein product of the RB1 gene, pRB, while sT interacts with MYC, resulting in TP53 inactivation. Unlike MCPyV-positive MCCs, virus-negative MCCs are characterized by a high mutational burden induced by ultraviolet (UV) exposure [1, 2]. Routine clinical practice differentiates therapeutic approaches for MCC based on tumor staging, subdividing it into primary and advanced/metastatic entities that receive specific treatments. Surgery is considered the standard approach for patients with local/regional MCC, followed by adjuvant radiotherapy. Although MCC is considered a chemosensitive tumor, adjuvant chemotherapy has shown poor durability and is not recommended. Definitive radiotherapy, applied in inoperable cases, is associated with a high risk of local recurrence and metastasis [3]. Systemic chemotherapy has traditionally been used to treat metastatic and recurrent MCC, but with low response rates. Recommendations for systemic therapy of advanced/metastatic MCC have been largely based on data from tumors with similar neuroendocrine features, such as small-cell lung carcinoma [4]. The increased understanding of MCC biology, along with the documented high immunogenicity of MCC and expression of programmed death ligand-1 (PD-L1) in tumors, has prompted the development of immunotherapies relying on programmed death-1/ligand-1 inhibitors (anti-PD-(L)1), which have shown promising clinical efficacy. Therapies with avelumab (anti-PD-L1), pembrolizumab, and nivolumab (both anti-PD-1) are indeed effective for treating advanced/metastatic MCC [5]. Multiple ICI-based clinical trials and real-world evidence indicate improved response rates in advanced/metastatic MCC patients receiving first-line immunotherapy compared with those undergoing chemotherapy [3, 6]. Moreover, the treatment of advanced MCC patients who develop resistance to anti-PD-(L)1 has demonstrated worsened efficacy when patients were treated with further chemotherapy compared to anti-PD-(L)1 rechallenge [3]. Notably, although displaying unique molecular traits, routine clinical practice does not differentiate primary MCPyV-positive and virus-negative MCCs from a therapeutic perspective [4]. Identifying the cellular origin of MCC, a topic of ongoing research and debate, is crucial for developing novel preclinical models, ultimately enhancing the clinical management of the disease.

## The Traditional Merkel Cell Origin Theory

Merkel cells (MCs), skin mechanoreceptors, have traditionally been considered the MCC origin cells due to morphological similarities with MCC cells. However, this theory has been gradually questioned since (i) MCs are primarily located in the basal layer of the epidermis and in the adnexa, specifically hair follicles, while MCC is a dermal/subcuticular tumor, (ii) CK20 expression patterns differ sharply between MCs and MCC cells, with the presence of loosely and diffusely arranged filaments in MCs and whirl- or plaque-like aggregates in MCC cells [7] (iii) unlike MCs, MCC cells express CD171 and CD24 and c-kit, (iv) no benign MC tumors or collision tumors with MCCs have been identified, and (v) MCs are terminally differentiated, making them unlikely for MCPyV infection [1].

## Alternative Origin Cell Types

**Fig. 1:**
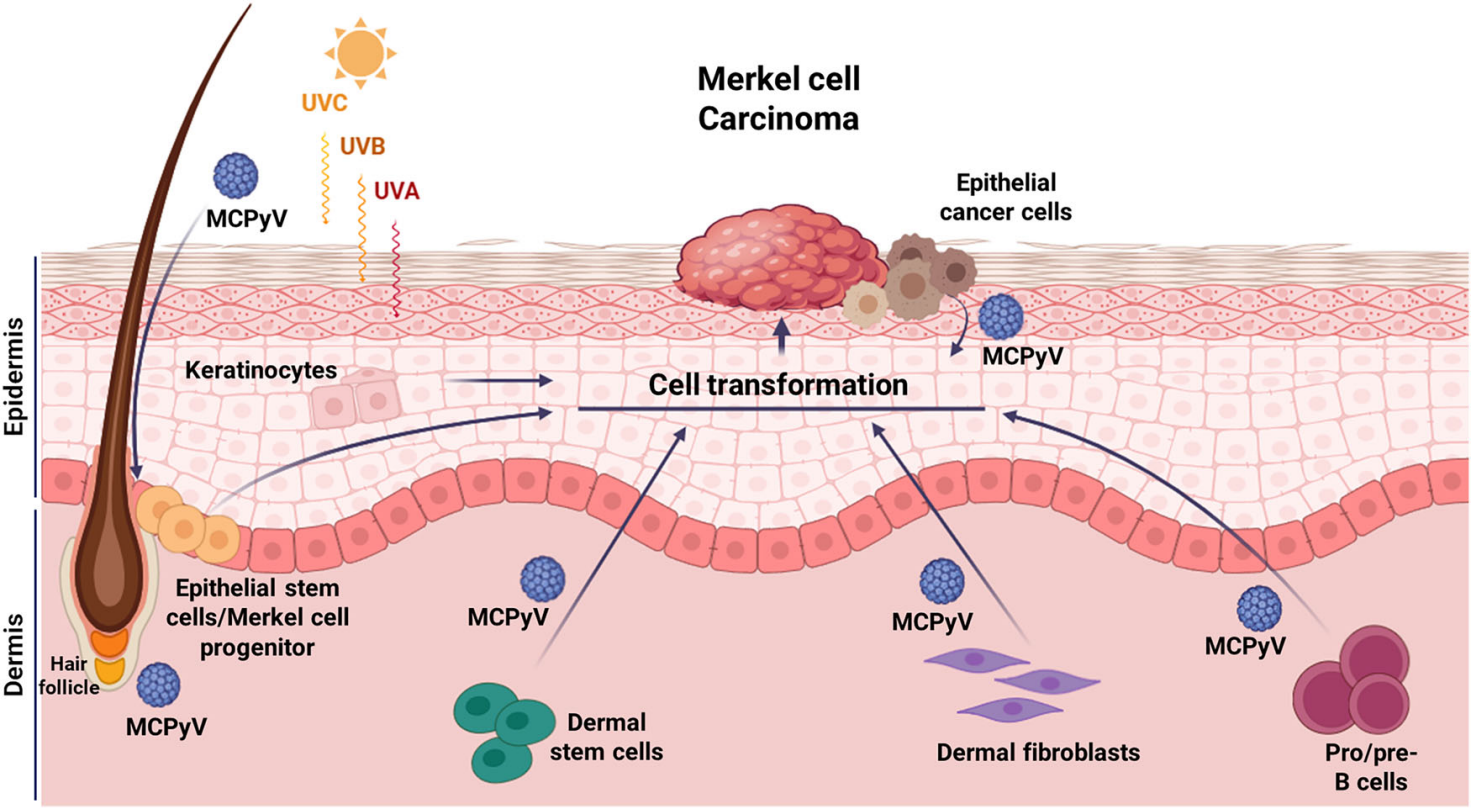
Graphical representation of the proposed cells of origin of Merkel cell carcinoma (MCC). Hypothetical MCC origin cells include epithelial stem cells/Merkel cell progenitors, keratinocytes, epithelial cancer cells, dermal stem cells, dermal fibroblasts, and pro/pre-B cells. Merkel cells have been initially considered the original cells of MCC. However, since Merkel cells are terminally differentiated and post-mitotic, this hypothesis has been questioned. Typically located in the basal layer of the epidermis, Merkel cells are likely derived from epidermal or hair follicle stem cells. Epidermal stem cells/Merkel progenitors have been suggested as an alternative cell of origin. Another hypothesis is that MCC derives from non-epidermal origin such as dermal stem cells, dermal fibroblasts, or pro/pre-B cells, residing in the dermal layer, due to the presence of neuronal cell markers, the ability to support Merkel cell polyomavirus (MCPyV) infection and the expression of B-lymphoid markers, respectively. However, dermal stem cells, fibroblasts and pro/pre-B cells, which are shielded from significant ultraviolet (UV) A, B and C, exposure, are unlikely to be the origin of UV-associated MCC. UV-associated MCCs have been proposed to arise from epidermal keratinocytes, which are more likely to be exposed to UV radiation. Alternatively, epithelial cancer cells, originating other skin tumors, such as squamous cell carcinoma, may transform into MCC cells, acquiring a neuroendocrine phenotype in the process.

The trilinear differentiation of MCC, characterized by neuroendocrine, epithelial, and B-lymphoid lineage markers, motivates the debate [8]. Alternative origin cell types have therefore been proposed, including pro-/pre-B and dermal stem cells, hair follicle/interfollicular epidermic cells, dermal fibroblasts and epidermal keratinocytes (Fig. 1) [1]. The argument for pro-/pre-B cells is supported by histological similarities between lymphoid malignancies and MCC, the expression of B-lymphoid markers in MCC and the detection of the MCPyV genome in chronic lymphoid leukemia cells [9, 10]. The dermal stem cell theory relies on the early identification of putative stem cells in MCC tumors [11–14]. Although both B cells and dermal stem cells can explain the typical dermal presentation of MCC tumors, MCPyV DNA has not been identified in these cell types to date. Additionally, it is unlikely that cells located in the dermis can acquire UV-induced point mutations characterizing MCPyV-negative MCCs.

The presence of two etiologies suggests MCC may originate from distinct cell types. MCPyV-positive MCCs might derive from dermal fibroblasts, while MCPyV-negative MCCs from keratinocytes/epidermal precursor cells. The absence of mutations in MCPyV-positive tumors supports the dermal fibroblast theory, as it is unlikely that these tumors originate from epidermal sun-exposed cells. Moreover, while keratinocytes and fibroblasts have been suggested as targets for MCPyV infection, in vitro evidence indicates dermal fibroblasts as the sole cells supporting viral transcription/replication [11]. Dermal fibroblasts may therefore be the MCPyV-positive MCC originating cells. However, since the post-viral infection/transformation mechanisms are unknown and MCPyV footprints have never been detected in dermal fibroblasts, this hypothesis remains unfeasible.

## The Epithelial Origin Theory

The epithelial origin theory is gaining attention for the presence of comparable UV mutational signatures between MCPyV-negative MCCs and epidermal tumors, including squamous cell carcinoma. Moreover, MCs originate from epithelial stem cells during embryonic development [11, 15, 16]. As MCC cells present a specific neuroendocrine gene expression pattern and phenotype, one could argue that these tumor cells might originate from the same epidermal lineage as MCs. In this context, understanding the molecular processes that drive MC development would provide insights into the MCC originating cell. Two transcription factors, GLI1 and ATOH1, identified as lineage-dependent oncogenes in MCC [12, 17], play distinct roles during MC development. ATOH1 plays a pivotal role in MC differentiation and is expressed in mature MCs, while GLI1 expression is observed in MC precursors [18, 19]. Co-expression of GLI1 and MCPyV oncoproteins in a human keratinocyte cell model has been reported to prompt these cells to express specific MC markers and to exhibit a typical MC phenotype, which was favored by the inhibitory activity of MCPyV LT on ATOH1 degradation [20]. A recent study employed a lineage tracing to identify epidermal cells expressing SOX9 (SOX9^+^) as MC progenitors in postnatal murine skin, while MCPyV LT/sT expression enforced neuroendocrine and MC lineage reprogramming in these cells. Notably, the expression of GLI1 has been reported in SOX9^+^ cells [20], supporting the hypothesis that these epidermal cells function as MC progenitors. Hence, MCPyV-driven MCC might originate from epidermal progenitors transformed by MCPyV [21]. In addition, the expression of MCPyV LT in ATOH1-reprogrammed epidermal cells and their neuroendocrine progeny can drive the formation of hair follicle-derived MCCs in vivo, accompanied by the loss of p53, as occurring in MCPyV-positive MCCs [22]. A recent study conducted in MCC cells investigated key components of the Hippo signaling pathway, notably YAP1 and its co-regulator WWTR1, which controls stem cell renewal and differentiation [23]. The study reported an inverse correlation between a neuroendocrine gene signature and YAP1 and WWTR1 expressions in MCC cells. Moreover, the expression of either YAP1 or WWTR1 in MCPyV-positive MCC cells induces cell cycle arrest, at least in part, through the TEAD-dependent transcriptional repression of MCPyV LT. Consistently, loss of YAP1 expression has been documented in MCPyV-positive MCCs [24]. The requirement for MCPyV LT expression may therefore drive reduced levels of YAP1 and WWTR1 in MCPyV-positive MCC [23]. These studies collectively suggest that, during MCC initiation, phenotypic changes, including the acquisition of neuroendocrine characteristics, occur during the transformation of a stem cell from the epidermal lineage.

Another critical aspect supporting the epithelial origin theory is the documented occurrence of collision tumors involving MCCs and squamous cell carcinomas [25, 26]. Hence, the molecular characterization of such collision tumors could be crucial for acquiring relevant information on the MCC biology. Kervarrec and colleagues identified a mutational overlap between the two components of a collision tumor consisting of a trichoblastoma, a benign tumor harboring hair follicle differentiation, derived from the epithelial lineage, and a MCPyV-positive MCC. These findings support the hypothesis that MCPyV-positive MCCs might derive from hair follicle cells. In another study, the same group identified numerous shared mutations between combined MCCs and squamous cell carcinomas in situ [25]. In line with these findings, Harms et al. documented the clonal relatedness between collision tumors consisting of MCCs and squamous cell carcinomas in situ. Notably, loss of Rb protein expression, which has also been observed in the squamous cell carcinoma component [25, 27], distinctive transcriptional profiles, as well as decreased global H3K27Me3, accompanied the shift from squamous towards a neuroendocrine morphology in MCCs [26]. These findings collectively support the potential epithelial origin of MCC. MCPyV integration likely occurs in an epithelial precursor cell prior to MCPyV-driven clonal expansion, while the same originating cell type may undergo a specific molecular switch that drives neuroendocrine differentiation, leading to UV-mutated, MCPyV-negative MCCs. As a support, Gravemeyer et al described minor variations in the DNA methylome between MCPyV-positive and -negative MCCs, which were attributed to distinct MCPyV-driven and UV-associated carcinogenetic processes rather than different origin cells. Moreover, by applying a scoring model for epithelial and neuroendocrine characteristics, both MCPyV-positive and -negative MCCs clearly scored as epithelial cancers [28]. Notably, the pivotal phenotypic characteristic of MCC regardless of its viral or UV-associated carcinogenesis result from neuroendocrine differentiation. Although the specific oncogenic driver contributing to the neuroendocrine transition in MCC is unknown, specific epigenetic changes, such as histone-posttranslational modifications, are currently considered primary events involved [26, 29, 30]. Hair follicle stem cells could possess the necessary epigenetic plasticity to express neuroendocrine markers and acquire a MC-like phenotype either in the presence of MCPyV or as a consequence of UV-associated mutations and/or dysregulation of epigenetic pathways.

## Unresolved Questions

Significant recent advances in MCC biology are enhancing our understanding of the mechanisms driving tumor initiation and bringing us closer to identifying its cellular ancestry. With the exclusion of MCs as MCC origin cells, some evidence suggested that both MCPyV-negative and -positive MCCs might represent distinct entities originating from specific cells residing in different regions of the human skin [13]. In this model, MCC might result from convergent tumor evolution, due to the differentiation of two origin cells that, upon transformation, exhibit a neuroendocrine phenotype. Besides this model, there is compelling genetic and epigenetic evidence suggesting that both MCC subsets might derive from the same undifferentiated epithelial precursor cell, possibly a hair follicle cell, which acquires a MC-like phenotype, while undertaking distinct MCPyV-driven and UV-associated transformative events. In this regard, some important questions remain to be answered. For instance, why has the presence of MCPyV footprints never been demonstrated in epithelial precursor cells of human skin? What is the molecular mechanism driving neuroendocrine differentiation in these cells, regardless of viral status? Moreover, how can these cells express B cell specific markers during transformation as occurring in MCC cells? Another critical aspect that further complicates matters is the intriguing puzzle surrounding the still unknown relationship among human cells that support a natural MCPyV life cycle, their post-infection fate, and those undergoing MCPyV-driven transformation, leading to MCC. If the fibroblast theory is correct, what biological events lead to the generation of a transformed cell with a specific neuroendocrine phenotype? While the potential of fibroblasts to acquire a Merkel-cell-like phenotype has not yet been demonstrated, experimental evidence supports this phenomenon in keratinocytes and particularly MC precursors, which are now known to derive from the epidermal lineage. In this regard, how can MCPyV reach dermal fibroblasts and/or MC precursors? MCPyV presumably reaches the dermal compartment either as a consequence of microabrasions, as performed by other DNA tumor viruses such as human papillomaviruses [31, 32], or throughout hair follicles where hair follicle stem cells reside. In this regard, an alternative reported theory implies that upon infecting dermal fibroblasts, MCPyV could occasionally enter undifferentiated MC precursors located closely, either within the epidermal basal layer or inside hair follicles [1]. Regarding virus-negative MCC, the molecular switch, potentially involving epigenetic modifications, that enables a possible epithelial precursor cell harboring UV-associated mutations to acquire the neuroendocrine phenotype, is yet to be identified. Both hypotheses still require validation, including the identification of (i) MCPyV in dermal fibroblasts and/or epithelial stem cells in human skin, and (ii) the molecular players driving neuroendocrine differentiation in virus-negative MCC.

Identifying the MCC-originating cell is crucial for developing novel preclinical models for advancing therapeutic strategies. The acquisition of a neuroendocrine phenotype by an epithelial precursor cell, either *via* MCPyV- or UV-associated transformative events, may lead to distinct MCC subtypes with differing clinical behaviors. In this context, should the two MCC subtypes be considered separate entities that clinically behave differently and thus receive distinct therapeutic approaches? This hypothesis could enable the stratification of primary MCPyV-positive and virus-negative MCCs, which are currently not differentiated from a therapeutic perspective [4]. Notably, growing evidence indicates that virus-negative MCCs are more aggressive, with a higher risk of disease progression and metastasis, leading to poorer outcomes, compared to MCPyV-positive MCCs [33]. The observation that MCC comprises two biologically and clinically distinct subtypes therefore represents an option worth exploring.

## Conclusions

In conclusion, recent findings suggest an epithelial origin for MCC. While numerous aspects have been uncovered, further in vivo research using transgenic mice is necessary to accurately recapitulate MCC and validate these hypotheses. Identifying the origin cell of MCC will undoubtedly lead to the development of more accurate tumor models that better recapitulate MCC biology, significantly advancing clinical applications. Moreover, a better understanding of the cellular ancestry of MCC could provide insights into identifying risk factors and developing preventive strategies to minimize the risk of this lethal neuroendocrine skin neoplasm.
